# Trends in mortality from nutritional and metabolic diseases in the United States (1999–2020): An epidemiologic analysis using CDC WONDER

**DOI:** 10.1097/MD.0000000000048928

**Published:** 2026-05-15

**Authors:** Juan He, Xianghua Shuai, Hana Zhu, Keran Xia

**Affiliations:** a Zhejiang Chinese Medical University,Hangzhou, China; b Department of Pediatrics, The First People’s Hospital of Yuhang District, Hangzhou, China.

**Keywords:** CDC WONDER, mortality, nutritional and metabolic diseases, the United States

## Abstract

Nutritional, and metabolic diseases are a major public health issue in the United States, affecting population health significantly. This study aims to analyze the mortality trends of these diseases and related demographic disparities in the US from 1999 to 2020. Data on deaths from nutritional, and metabolic diseases in the US (1999–2020) and demographic characteristics (sex, census region, race, urbanization, and age group) were collected. Age-adjusted mortality rates (AAMRs) were calculated, with crude mortality rates used as a substitute for AAMR in age group analyses. The average annual percent change (AAPC) with 95% confidence intervals (CIs) was estimated to quantify trends, with *P* < .05 considered statistically significant. From 1999 to 2020, the total number of deaths from these diseases in the US decreased by 25.04% (from 631 to 473), with an overall AAMR dropping from 1.04 (95% CI: 0.96–1.12) to 0.80 (95% CI: 0.72–0.87) and an AAPC of −1.71% (95% CI: −2.16 to −1.26%, *P* < .05). Significant declines were observed across sex (females: −28.77%, AAPC = −1.82%; males: −21.83%, AAPC = −1.77%), census regions (Midwest with the largest decrease: −33.81%, AAPC = −1.21%), urbanization levels (metropolitan: −25.62%, AAPC = −1.81%; nonmetropolitan: −22.12%, AAPC = −1.49%), and age groups (<1 year with the largest decrease: −43.68%, AAPC = −2.66%). By race, Non-Hispanic (NH) White individuals had the most substantial death reduction (−40.66%, AAPC = −2.06%), while Hispanic (20.24%) and NH Other (47.83%) groups saw increased deaths; NH Black individuals had a 19.53% decrease (AAPC = −1.49%), and the AAPC for NH Other was nonsignificant (−0.51%, *P* > .05). Mortality from nutritional, and metabolic diseases in the US showed a significant downward trend from 1999 to 2020, but demographic disparities persisted across sex, region, race, urbanization, and age groups. Targeted interventions for high-risk subgroups are essential to further reduce mortality and eliminate disparities.

## 1. Introduction

Nutritional, and metabolic diseases encompass a broad spectrum of conditions – including diabetes mellitus, thyroid disorders, obesity-related complications, and inborn errors of metabolism – that collectively pose a substantial burden on public health in the United States.^[1–3]^ These diseases not only impair the quality of life of affected individuals but also contribute to long-term morbidity, healthcare system strain, and premature mortality, with far-reaching implications for population health equity and socioeconomic stability. Over the past 2 decades, advancements in medical care, improved access to diagnostic tools, enhanced public health awareness, and targeted interventions for chronic metabolic conditions have reshaped the landscape of disease management. However, the extent to which these advancements have translated into reduced mortality, and whether such improvements are equitably distributed across diverse demographic groups, remains a critical area of inquiry.

Demographic disparities in health outcomes have long been a persistent challenge in the U.S. healthcare system, with differences in mortality rates observed across sex, racial/ethnic groups, geographic regions, urban–rural divides, and age strata.^[4–6]^ For nutritional, and metabolic diseases, these disparities may stem from a complex interplay of factors, including access to healthcare services, socioeconomic status, health literacy, cultural differences in disease management, environmental exposures, and genetic predispositions. For instance, racial and ethnic minority groups have historically faced higher burdens of diabetes and obesity, while rural populations often encounter barriers to specialized care for chronic metabolic conditions. For instance, the fact that infants and young children are more prone to metabolic complications underscores the critical need for interventions tailored to their age. Understanding how mortality trends for these diseases have evolved across these demographic dimensions is essential to identifying unmet needs and guiding the development of equitable public health strategies.

While prior studies have documented trends in specific metabolic conditions (e.g., diabetes-related mortality), comprehensive analyses of overall mortality from nutritional, and metabolic diseases – encompassing the full range of related conditions and examining multiple demographic disparities simultaneously – remain limited. This gap in knowledge hinders the ability to assess the overall effectiveness of public health and clinical interventions and to address persistent inequities. To fill this void, the present study aims to analyze mortality data from nutritional, and metabolic diseases in the United States over a 22-year period (1999–2020). By evaluating trends in the total number of deaths, age-adjusted mortality rates (AAMRs), and average annual percent changes (AAPC) across sex, census regions, race/ethnicity, urbanization levels, and age groups, this study seeks to provide a holistic understanding of how mortality from these diseases has changed over time and to identify subgroups that may require targeted interventions. The findings of this study will inform public health policies, clinical practice guidelines, and resource allocation to reduce mortality and advance health equity for all U.S. populations affected by nutritional, and metabolic diseases.

## 2. Methods

This retrospective analytic epidemiological study was conducted to analyze mortality trends from nutritional and metabolic diseases in the United States over a 22-year period (1999–2020). Data were sourced from the Centers for Disease Control and Prevention’s Wide-ranging Online Data for Epidemiologic Research (CDC WONDER) database, a publicly accessible repository of de-identified nationwide health statistics that supports population-level epidemiological analyses. Specifically, the Multiple Cause-of-Death Public Use Record files were utilized to identify deaths where nutritional or metabolic diseases were listed as the underlying cause, in accordance with the International Classification of Diseases coding standards corresponding to the study period (ICD-10 Codes: E00–E88 for nutritional and metabolic diseases). Institutional review board approval was waived as the database contains de-identified data and the study adheres to the STROBE reporting guidelines.^[7–9]^

The study population included all individuals with deaths recorded in the CDC WONDER database between 1999 and 2020 that met the aforementioned cause-of-death criteria, with no age restrictions to ensure the inclusion of all age strata (from < 1 year to older age groups). Population denominator data for mortality rate calculations were obtained from the U.S. Census Bureau’s annual population estimates, and were stratified by the same demographic variables as the mortality data to ensure consistency in subgroup analyses. Mortality data were further stratified by key demographic and geographic variables to assess disparities, including: sex (male and female); census region (Northeast, Midwest, South, and West, as defined by the U.S. Census Bureau); race/ethnicity (Hispanic, non-Hispanic [NH] White, NH Black, and NH Other [including American Indian/Alaskan Native, Asian/Pacific Islander, and other racial groups]); urbanization level (Metropolitan [large, medium, and small metropolitan areas with population ≥ 50,000] and Nonmetropolitan [counties with population < 50,000], based on the National Center for Health Statistics 2013 Urban–Rural Classification Scheme for Counties)^[10]^; and age groups (<1 year, 1–4 years, and 5–14 years).

Two primary mortality rate metrics were calculated for this analysis: AAMRs per 100,000 population for all stratifications except age groups, which were standardized to the 2000 U.S. standard population to account for age-related differences in population structure across time and subgroups; crude mortality rates (also reported per 100,000 population), which were used as a substitute for AAMRs in age group analyses due to age-specific data limitations.

Temporal trends in mortality rates were analyzed using the Joinpoint Regression Program (Version 4.9.0.0, National Cancer Institute), the dedicated statistical software for this study. The AAPC and 95% confidence intervals (CIs) were estimated to quantify the magnitude and direction of mortality trends over the entire study period; where significant changes in trend direction were observed, annual percent changes were additionally calculated for distinct time segments. Statistical significance was defined as a two-sided *P* value < .05 for all trend and rate analyses.

## 3. Results

### 3.1. Overall mortality trends

This study set out to analyze the temporal mortality trends of nutritional and metabolic diseases in the United States from 1999 to 2020 and to examine persistent disparities in these outcomes across key demographic and geographic subgroups, including sex, U.S. census region, race/ethnicity, urbanization level, and age group (under 15 years). Between 1999 and 2020, a total of 473 deaths from nutritional, and metabolic diseases were recorded in the United States, representing a 25.04% decrease from the 631 deaths reported in 1999. The overall AAMR declined significantly from 1.04 (95% CI: 0.96–1.12) per 100,000 population in 1999 to 0.80 (95% CI: 0.72–0.87) in 2020, with an AAPC of −1.71% (95% CI: −2.16 to −1.26%, *P* < .05; Table 1).

**Table 1:** Endocrine, nutritional and metabolic deaths and AAMR in the United States from 1999 to 2020 and their changing trends.

Characteristic	Deaths			AAMR		
	1999	2020	Percent change (%)	1999 (95% CI)	2020 (95% CI)	AAPC (95% CI)
Both	631	473	−25.04	1.04 (0.96 to 1.12)	0.80 (0.72 to 0.87)	−1.71 (−2.16 to −1.26)*
Sex						
Female	292	208	−28.77	0.99 (0.88 to 1.10)	0.70 (0.61 to 0.80)	−1.82 (−2.34 to −1.30)*
Male	339	265	−21.83	1.09 (0.98 to 1.21)	0.89 (0.78 to 1.00)	−1.77 (−2.22 to −1.33)*
Census region						
Northeast	103	72	−30.10	0.99 (0.80 to 1.18)	0.73 (0.57 to 0.91)	−2.22 (−3.00 to −1.44)*
Midwest	139	92	−33.81	1.02 (0.85 to 1.19)	0.74 (0.60 to 0.91)	−1.21 (−2.25 to −0.17)*
South	260	217	−16.54	1.20 (1.05 to 1.34)	0.93 (0.80 to 1.05)	−1.79 (−2.29 to −1.28)*
West	129	92	−28.68	0.89 (0.73 to 1.04)	0.62 (0.50 to 0.75)	−2.11 (−2.69 to −1.52)*
Race						
Hispanic	84	101	20.24	0.75 (0.60 to 0.93)	0.67 (0.54 to 0.80)	−1.37 (−1.99 to −0.75)*
NH Black	128	103	−19.53	1.41 (1.17 to 1.66)	1.10 (0.89 to 1.31)	−1.49 (−2.43 to −0.54)*
NH White	391	232	−40.66	1.08 (0.97 to 1.18)	0.77 (0.67 to 0.87)	−2.06 (−2.55 to −1.56)*
NH Other	23	34	47.83	0.81 (0.51 to 1.21)	0.74 (0.51 to 1.04)	−0.51 (−1.71 to 0.70)
Urbanization						
Metropolitan	527	392	−25.62	1.03 (0.94 to 1.12)	0.79 (0.71 to 0.87)	−1.81 (−2.34 to −1.29)*
Nonmetropolitan	104	81	−22.12	1.19 (0.96 to 1.42)	1.04 (0.83 to 1.29)	−1.49 (−2.33 to −0.64)*
Age groups†						
< 1 yr	261	147	−43.68	6.88 (6.04 to 7.71)	3.94 (3.30 to 4.57)	−2.66 (−3.21 to −2.10)*
1–4 yrs	153	124	−18.95	1.00 (0.84 to 1.16)	0.80 (0.66 to 0.94)	−1.93 (−2.62 to −1.22)*
5–14 yrs	217	202	−6.91	0.53 (0.46 to 0.60)	0.49 (0.42 to 0.56)	−0.85 (−1.35 to −0.34)*

AAMR = age-adjusted mortality rate, AAPC = average annual percent change, CI = confidence interval, NH = non-Hispanic.

* *P* < .05.

† For the age groups, the crude mortality rate was used as a substitute for AAMR, and the AAPC was computed based on the crude mortality rate.

### 3.2. Mortality by sex

**Figure 1.**
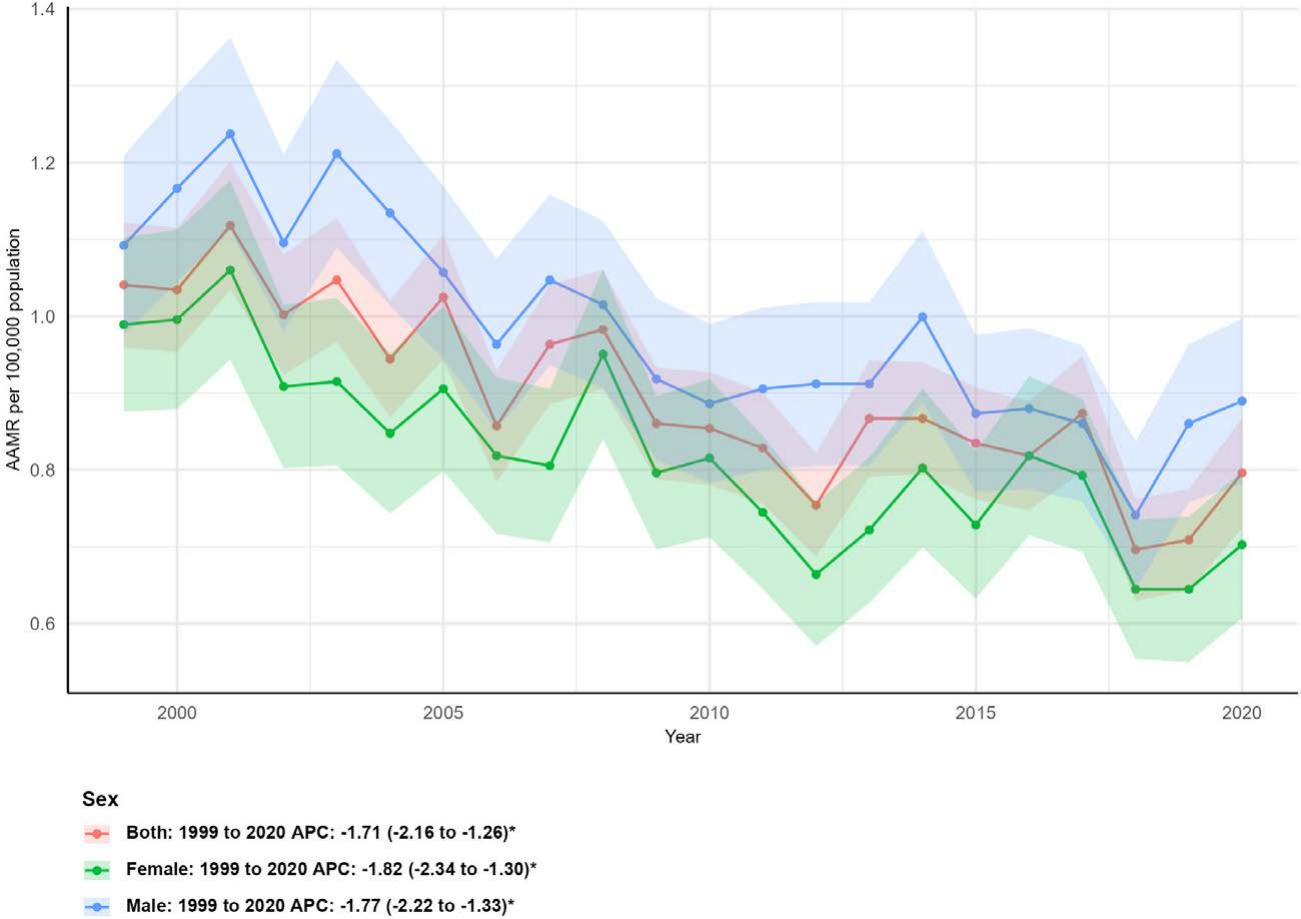
Mortality trends by sex. AAMR = age-adjusted mortality rate, APC = annual percent change.

Mortality rates decreased for both sexes over the study period. Female deaths fell by 28.77% (from 292 to 208), with an AAMR declining from 0.99 (95% CI: 0.88–1.10) to 0.70 (95% CI: 0.61–0.80) and an AAPC of −1.82% (95% CI: −2.34 to −1.30%, *P* < .05). Male deaths decreased by 21.83% (from 339 to 265), with an AAMR dropping from 1.09 (95% CI: 0.98–1.21) to 0.89 (95% CI: 0.78–1.00) and an AAPC of −1.77% (95% CI: −2.22 to −1.33%, *P* < .05; Fig. 1).

### 3.3. Mortality by census region

**Figure 2.**
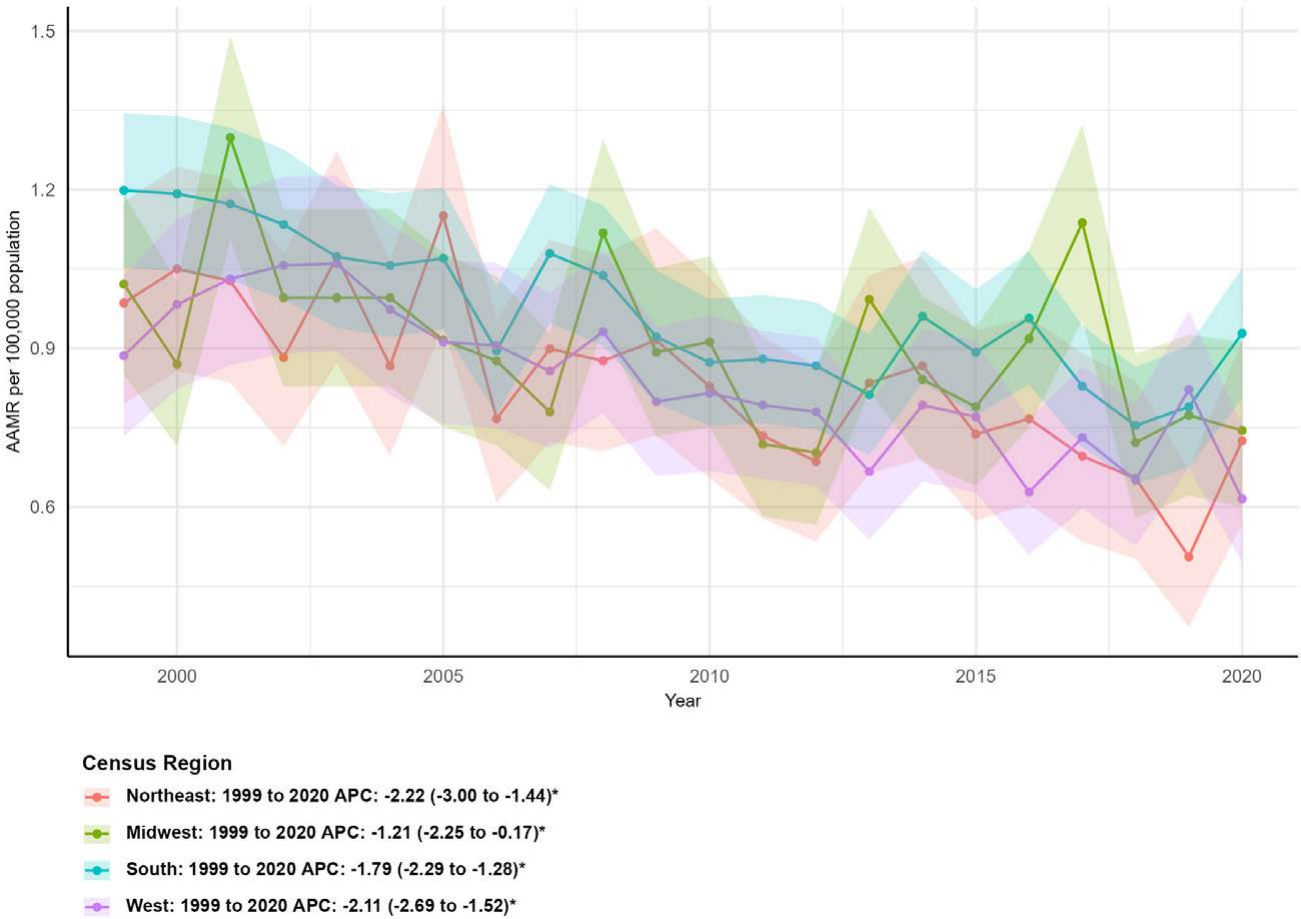
Mortality trends by census region. AAMR = age-adjusted mortality rate, APC = annual percent change.

All 4 U.S. census regions exhibited significant declines in mortality. The Midwest had the largest percentage decrease in deaths (−33.81%, from 139 to 92), with an AAMR falling from 1.02 (95% CI: 0.85–1.19) to 0.74 (95% CI: 0.60–0.91) and an AAPC of −1.21% (95% CI: −2.25 to −0.17%, *P* < .05). The Northeast (−30.10%), West (−28.68%), and South (−16.54%) also showed notable reductions, with AAPCs of −2.22% (95% CI: −3.00 to −1.44%, *P* < .05), −2.11% (95% CI: −2.69 to −1.52%, *P* < .05), and −1.79% (95% CI: −2.29 to −1.28%, *P* < .05), respectively (Fig. 2).

### 3.4. Mortality by race/ethnicity

**Figure 3.**
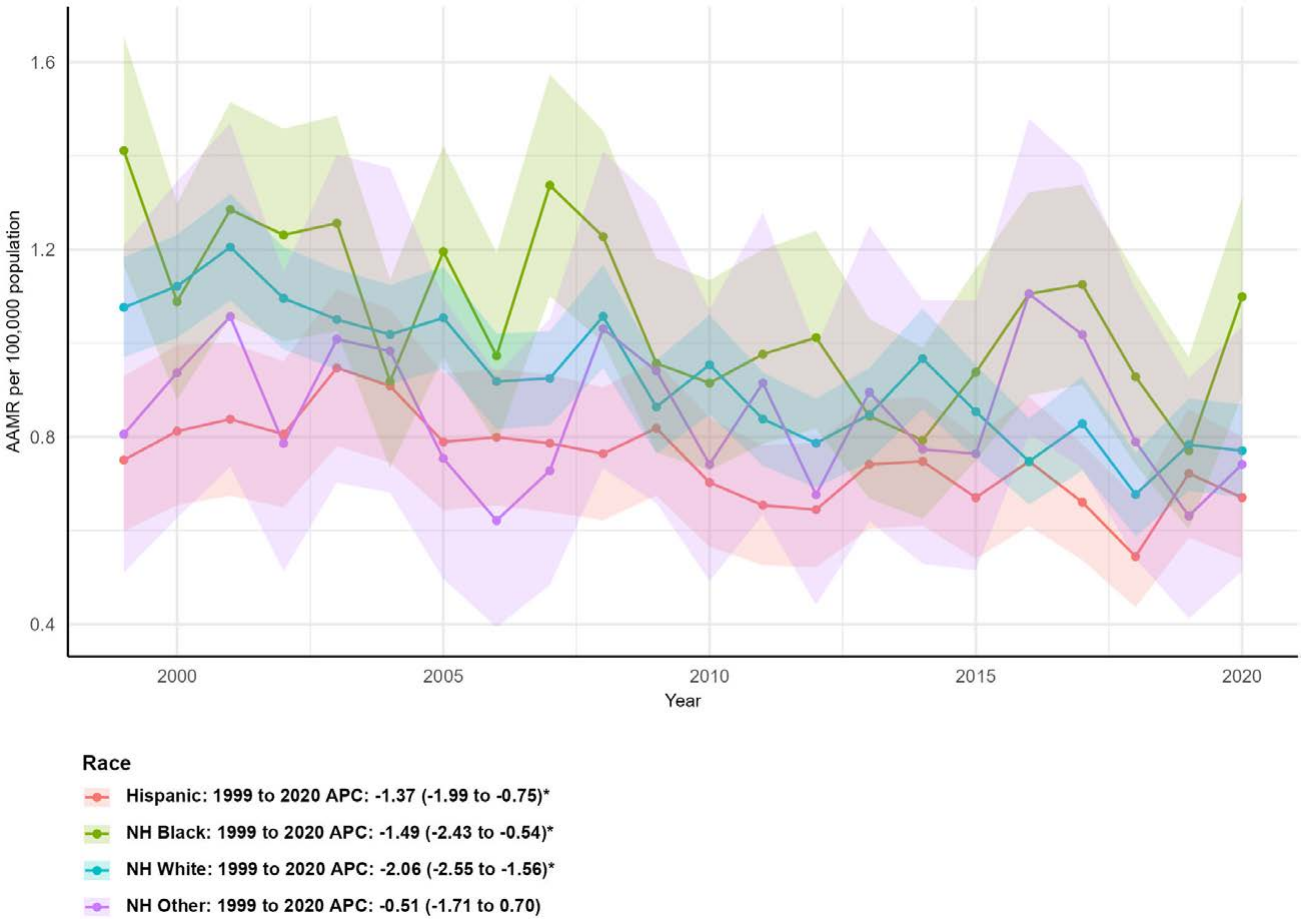
Mortality trends by race/ethnicity. AAMR = age-adjusted mortality rate, APC = annual percent change.

Racial/ethnic groups showed divergent trends. NH White individuals had the largest reduction in deaths (−40.66%, from 391 to 232), with an AAMR declining from 1.08 (95% CI: 0.97–1.18) to 0.77 (95% CI: 0.67–0.87) and an AAPC of −2.06% (95% CI: −2.55 to −1.56%, *P* < .05). NH Black deaths decreased by 19.53% (from 128 to 103), with an AAMR of 1.41 (95% CI: 1.17–1.66) in 1999 dropping to 1.10 (95% CI: 0.89–1.31) in 2020 and an AAPC of −1.49% (95% CI: −2.43 to −0.54%, *P* < .05). In contrast, Hispanic deaths increased by 20.24% (from 84 to 101) and NH Other deaths rose by 47.83% (from 23 to 34), with the latter’s AAPC (−0.51%, 95% CI: −1.71 to 0.70%) not reaching statistical significance (*P* > .05; Fig. 3).

### 3.5. Mortality by urbanization level

**Figure 4.**
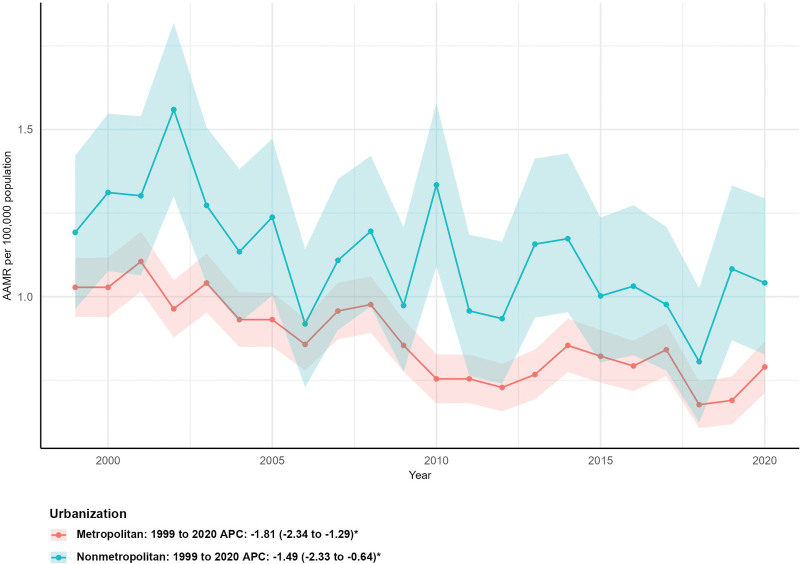
Mortality trends by urbanization level. AAMR = age-adjusted mortality rate, APC = annual percent change.

Both metropolitan and nonmetropolitan areas experienced mortality declines. Metropolitan areas saw a 25.62% reduction in deaths (from 527 to 392), with an AAMR falling from 1.03 (95% CI: 0.94–1.12) to 0.79 (95% CI: 0.71–0.87) and an AAPC of −1.81% (95% CI: −2.34 to −1.29%, *P* < .05). Nonmetropolitan areas had a 22.12% decrease (from 104 to 81), with an AAMR declining from 1.19 (95% CI: 0.96–1.42) to 1.04 (95% CI: 0.83–1.29) and an AAPC of −1.49% (95% CI: −2.33 to −0.64%, *P* < .05; Fig. 4).

### 3.6. Mortality by age group

**Figure 5.**
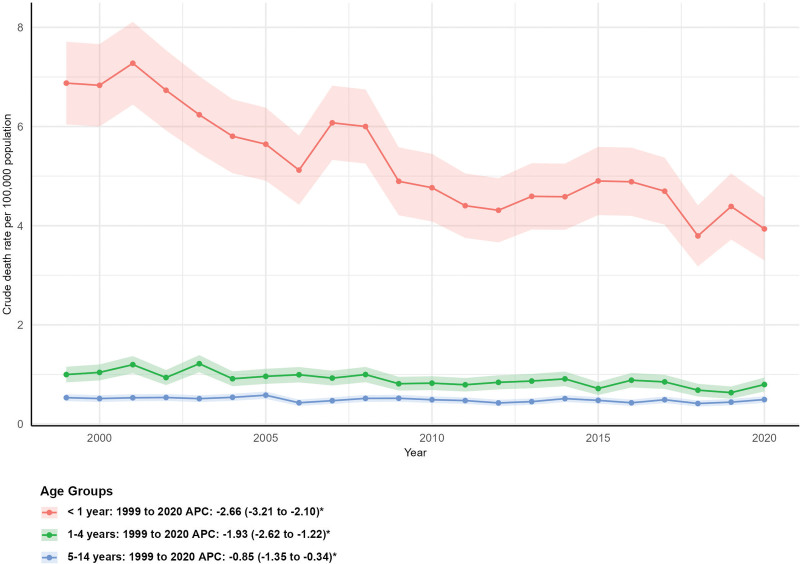
Mortality trends by age group. APC = annual percent change.

All age groups under 15 years showed significant declines in crude mortality rates. The < 1 year age group had the largest percentage decrease (−43.68%, from 261 to 147), with a crude mortality rate dropping from 6.88 (95% CI: 6.04–7.71) to 3.94 (95% CI: 3.30–4.57) and an AAPC of −2.66% (95% CI: −3.21 to −2.10%, *P* < .05). The 1 to 4 years age group saw an 18.95% reduction in deaths (from 153 to 124), with a crude mortality rate declining from 1.00 (95% CI: 0.84–1.16) to 0.80 (95% CI: 0.66–0.94) and an AAPC of −1.93% (95% CI: −2.62 to −1.22%, *P* < .05). The 5 to 14 years age group had the smallest decrease (−6.91%, from 217 to 202), with a crude mortality rate falling from 0.53 (95% CI: 0.46–0.60) to 0.49 (95% CI: 0.42–0.56) and an AAPC of −0.85% (95% CI: −1.35 to −0.34%, *P* < .05; Fig. 5).

## 4. Discussion

This study comprehensively analyzed mortality trends from nutritional, and metabolic diseases in the United States over a 22-year period (1999–2020), revealing a significant overall downward trend alongside persistent demographic and geographic disparities. The total number of deaths decreased by 25.04%, with an AAPC of −1.71% in AAMRs, reflecting progress in addressing these conditions while highlighting unmet needs in vulnerable subgroups.

The overall decline in mortality aligns with broader advancements in public health and clinical care over the study period. Improved access to diagnostic tools has enabled earlier detection of endocrine and metabolic disorders, such as diabetes and thyroid diseases, allowing for timely intervention.^[11,12]^ Progress in the clinical care and management of nutritional and metabolic disorders has further improved patient outcomes, supported by the establishment of standardized treatment protocols, enhanced pharmacotherapeutic strategies, and the expansion of evidence-based lifestyle intervention initiatives.^[13,14]^ Additionally, increased public health awareness campaigns targeting risk factors like obesity, poor nutrition, and sedentary behavior have likely reduced the incidence and severity of these diseases, further driving down mortality.^[15,16]^ These findings are consistent with prior research documenting reductions in mortality from chronic metabolic conditions amid broader healthcare system improvements.

Notable disparities emerged across demographic subgroups, underscoring the need for targeted interventions. Females experienced a slightly greater reduction in mortality (−28.77%) compared to males (−21.83%), which may relate to differences in healthcare-seeking behaviors, greater adherence to treatment regimens, or earlier recognition of symptoms. Among racial/ethnic groups, NH White individuals saw the largest death reduction (−40.66%), while Hispanic and NH Other populations experienced increases of 20.24% and 47.83%, respectively. These disparities may stem from structural barriers, including limited access to affordable healthcare, socioeconomic inequalities, and cultural differences in disease perception and management.^[17,18]^ NH Black individuals, despite a 19.53% decrease in deaths, maintained higher AAMRs throughout the study period, reflecting longstanding racial inequities in healthcare access and quality.

Geographic variations also highlight critical gaps in care delivery. The Midwest region recorded the largest percentage decrease in deaths (−33.81%) but, alongside other regions, still exhibited persistent mortality burdens, potentially influenced by regional differences in healthcare infrastructure and public health resources. Rural areas showed a smaller decline in mortality (−22.12%) compared to metropolitan areas (−25.62%), consistent with known challenges in rural healthcare – including physician shortages, limited access to specialized care, and socioeconomic disadvantages.^[19,20]^ These geographic disparities emphasize the need to strengthen healthcare delivery in underserved regions.

Age-specific trends revealed the most substantial mortality reduction in infants under 1 year (−43.68%), likely driven by improvements in neonatal care, early screening for inborn metabolic disorders, and enhanced maternal health initiatives. In contrast, the 5 to 14 years age group had the smallest decrease (−6.91%), suggesting that while pediatric metabolic conditions are relatively rare, targeted prevention and management strategies for this age group may be less robust.^[21–23]^ The use of crude mortality rates for age group analyses, as a substitute for AAMRs, ensures consistency with age-specific data limitations but should be considered when interpreting comparative trends across age strata.

Several limitations of this study warrant acknowledgments. The reliance on CDC WONDER data, while providing nationwide coverage, is subject to potential inaccuracies in death certificate coding and classification. Additionally, the study does not account for differences in disease severity, comorbidities, or treatment adherence, which may influence mortality outcomes. Data on specific subtypes of nutritional and metabolic diseases (e.g., type 2 diabetes and thyroid cancer) were not available, limiting insights into trends for individual conditions. Future research should address these gaps by incorporating clinical data on disease subtypes and patient-level factors.

Despite these limitations, this study offers valuable insights for public health policy and clinical practice. To further reduce mortality and eliminate disparities, targeted interventions should prioritize vulnerable subgroups – including racial/ethnic minorities, rural populations, and males. Strategies could include expanding access to affordable healthcare, enhancing culturally tailored health education, and strengthening preventive care programs in underserved regions. For pediatric populations, particularly those aged 5 to 14 years, increased focus on early lifestyle interventions and screening may help reduce long-term mortality risk. Additionally, ongoing monitoring of mortality trends is essential to evaluate the effectiveness of public health initiatives and adapt strategies to emerging needs.

## 5. Conclusion

### 5.1. Summary of main findings

From 1999 to 2020, mortality from nutritional and metabolic diseases in the U.S. declined significantly overall (25.04% drop in total deaths; AAPC = −1.71%, *P* < .05) across all subgroups, with notable disparities. Larger declines were seen in females, the Midwest, infants < 1 year and metropolitan areas; smaller declines in males, rural areas and children aged 5 to 14 years. By race/ethnicity, NH White had the biggest reduction (−40.66%), NH Black a modest drop (−19.53%), while Hispanic and NH Other saw increased deaths (20.24% and 47.83%, respectively).

### 5.2. Insights and implications

The overall decline proves the effectiveness of improved diagnostics, clinical care and public health interventions in the U.S. However, persistent demographic and geographic disparities reflect unequal access to these advances. Marginalized groups (racial/ethnic minorities, rural residents, and males) and children aged 5 to 14 years face a disproportionate disease burden due to limited healthcare access, socioeconomic inequities and lack of tailored interventions, meaning national progress has not achieved health equity.

### 5.3. Recommendations for future directions

We recommend developing culturally tailored healthcare and public health programs for racial/ethnic minorities, strengthening rural healthcare infrastructure to expand access to specialized metabolic care, designing age-specific prevention and management strategies for children aged 5 to 14 years, implementing targeted interventions for males to boost their healthcare-seeking behavior and treatment adherence, and conducting further research on disease subtypes and patient-level factors while continuously monitoring mortality trends to dynamically optimize relevant interventions.

## Author contributions

**Conceptualization:** Juan He.

**Formal analysis:** Juan He.

**Funding acquisition:** Juan He.

**Project administration:** Keran Xia.

**Software:** Juan He, Xianghua Shuai.

**Validation:** Keran Xia.

**Visualization:** Juan He, Keran Xia.

**Writing – review & editing:** Hana Zhu, Keran Xia.
